# Longitudinal Metabolomics and Lipidomics Analyses Reveal Alterations Associated with Envenoming by *Bothrops asper* and *Daboia russelii* in an Experimental Murine Model

**DOI:** 10.3390/toxins14100657

**Published:** 2022-09-23

**Authors:** Nishikant Wase, José María Gutiérrez, Alexandra Rucavado, Jay W. Fox

**Affiliations:** 1 School of Medicine, University of Virginia, Charlottesville, VA 22908, USA; 2 Instituto Clodomiro Picado, Facultad de Microbiología, Universidad de Costa Rica, San José 11501, Costa Rica

**Keywords:** metabolomics, lipidomics, snake venoms, *Bothrops asper*, *Daboia russellii*, envenoming

## Abstract

Longitudinal metabolomics and lipidomics analyses were carried out on the blood plasma of mice injected intramuscularly with venoms of the viperid species *Bothrops asper* or *Daboia russelii*. Blood samples were collected 1, 3, 6, and 24 h after venom injection, and a control group of non-envenomed mice was included. Significant perturbations in metabolomics and lipidomics were observed at 1, 3, and 6 h, while values returned close to those of control mice by 24 h, hence reflecting a transient pattern of metabolic disturbance. Both venoms induced significant changes in amino acids, as well as in several purines and pyrimidines, and in some metabolites of the tricarboxylic acid cycle. KEGG analysis of metabolic pathways that showed those with the greatest change included aminoacyl tRNA synthesis and amino acid biosynthesis and metabolism pathways. With regard to lipid metabolism, there was an increase in triglycerides and some acyl carnitines and a concomitant drop in the levels of some phospholipids. In addition, envenomed mice had higher levels of cortisol, heme, and some oxidative stress markers. The overall pattern of metabolic changes in envenomed mice bears similarities with the patterns described in several traumatic injuries, thus underscoring a metabolic response/adaptation to the injurious action of the venoms.

## 1. Introduction

Snakebite envenoming is a neglected tropical disease that kills and maims hundreds of thousands of people every year, especially in sub-Saharan Africa, Asia, and Latin America [1]. The pathological and pathophysiological manifestations of envenomings vary depending on the composition of the venoms, whose complexity has been unveiled by their proteomic characterization [2,3]. Owing to the wide spectrum of clinical manifestations of envenomings, a detailed understanding of the mechanisms of actions of venoms is paramount for improving treatment and for the development of novel therapeutic interventions.

Traditionally, the study of the pathophysiological alterations of envenomings has been focused on clinical observations, histological assessment of affected tissues and alterations in laboratory parameters. The effects of venoms have been mostly associated with the direct deleterious actions of toxins on cells and extracellular matrix. In the case of envenomings by snakes of the family Viperidae, phospholipases A_2_ (PLA_2_s), metalloproteinases (SVMPs) and serine proteinases (SVSPs) induce a complex tissue damage at the anatomical site of venom injection, together with systemic effects, i.e., bleeding, coagulopathies, hemodynamic alterations and, in some cases, acute kidney injury [1,4]. However, beyond this common syndromic pattern, there are variations in the pathophysiological profiles of viperid envenomings which need further characterization.

More recent developments have shown that in addition to the direct action of venoms in cells and the extracellular matrix, envenomings are associated with the onset of diverse endogenous processes related to the response of the organism to envenoming. These typically involve inflammatory pathways, with the synthesis or release of a wide spectrum of mediators, together with the generation of *damage-associated molecular patterns* (DAMPs) and reactive oxygen species in the affected tissues, which amplify the tissue response [5,6,7]. Thus, the combination of direct venom-induced toxic effects and endogenous processes generate a complex landscape which likely impacts the outcome of envenoming.

One aspect of the endogenous changes involved which has received little attention has to do with the systemic metabolic alterations occurring during envenoming. Understanding these metabolic changes may bring new facets of insight into the dynamics of this set of pathophysiological alterations, and could have diagnostic and therapeutic implications, as new biomarkers might arise from such studies. To this end, the application of metabolomics to the study of snakebite envenoming should provide valuable information for characterizing the metabolic fluctuations occurring in the course of envenoming. Metabolomics, as based on the use of mass spectrometry and other advanced analytical methods, provides high throughput qualitative and quantitative information of metabolites in biological systems [8]. These metabolites include low molecular mass molecules such as amino acids, lipids, nucleic acids, organic acids, carbohydrates, and peptides. It has become clear that metabolites play a wide variety of roles in physiological processes as signaling molecules, modulators of cellular processes, participation in immune responses and onset of deleterious effects of various types, among other actions [9] Thus, metabolomics could become a valuable analytical tool to better understand the dynamics of snakebite envenoming. There have been a few studies on the metabolomics of animals injected with venoms of snakes [10], honeybees [11], scorpions [12] and in human cases of envenomings by wasp stings [13].

Our groups have been investigating the effects induced by two viperid snake venoms which have a high public health impact in Asia and Latin America, i.e., *Daboia russelii* and *Bothrops asper,* respectively. Although these venoms induce common alterations, they also show differences in their actions. *B. asper* venom induces the archetypical pattern of local and systemic effects characteristic of the majority of viperid venoms, i.e., local necrosis, edema, blistering and hemorrhage, and systemic bleeding, coagulopathy, shock, and acute kidney injury [14,15,16]. In the case of *D. russelii* venom, in addition to some typical effects induced by viperid venoms, it inflicts unique disturbances such as a systemic vascular leakage syndrome associated with hemoconcentration and a high incidence of acute kidney injury [17]. Moreover, their action in the microvasculature differs, as *B. asper* venom predominantly induces microvessel damage and hemorrhage while *D. russelii* venom induces an increase in vascular permeability leading to hemoconcentration [18]. Furthermore, differences have been observed in the proteomic profile of extracellular matrix and cellular proteins in exudates collected from tissues injected with venoms, as well as in the subproteomes of inflammatory mediators along time [19].

To gain further understanding on the pathophysiology of envenomings by these two species in an experimental murine model, a combined untargeted metabolomic and lipidomics analysis was undertaken to explore the changes of a wide array of metabolites and lipid species in the blood serum at various time intervals after the local injection of venoms in muscle. Results highlight significant changes in many metabolites and lipids in the course of envenoming, particularly in the first six hours, underscoring a complex metabolic response of the organism to envenoming.

## 2. Results

### 2.1. Metabolic Changes in Envenoming as Determined with Untargeted Metabolomics

**Figure 1:**
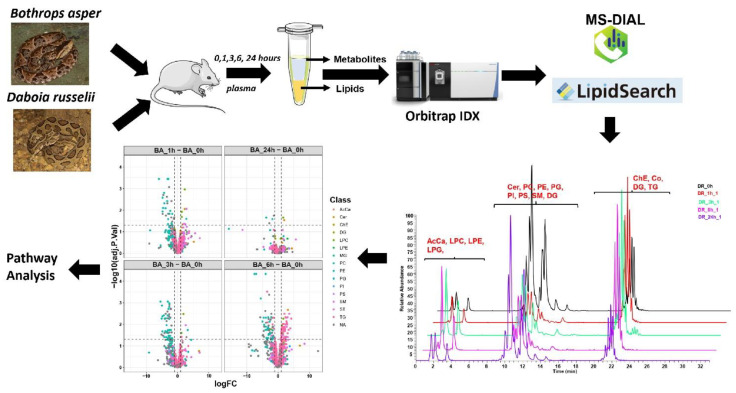
Experimental workflow for combined untargeted metabolomics and lipidomics analysis of plasma of mice injected with the venoms of *B. asper* and *D. russelii.* Control samples were obtained from non-envenomed mice.

Figure 1 depicts the experimental workflow for the combined untargeted metabolomics and lipidomics analysis carried out in this work. The changes in metabolites in the blood plasma of mice after intramuscular (i.m.) injection of the venoms of *B. asper* and *D. russelii* were assessed at four time intervals (1, 3, 6, and 24 h), as compared to non-envenomed controls. The resultant metabolite data matrix consisted of 15,600 spectral features in ESI (electrospray ionization)+ mode and 1594 spectral features in ESI− mode. We filtered the unknown peaks and peaks that have RSD > 30% in QC. A total of 631 and 106 in ESI+ and ESI−, respectively, were annotated by spectral search and by comparing the accurate mass and retention time to the in-house generated IORA library. The final data matrix had 380 metabolites that were confidently identified using accurate mass, retention time and MS/MS spectra (for in-house library) or with accurate precursor mass and spectral match using MS-DIAL and publicly available spectral libraries. Table 1 depicts the list of metabolites in plasma from envenomed mice showing the highest differences when compared to samples from non-envenomed mice.

**Figure 2:**
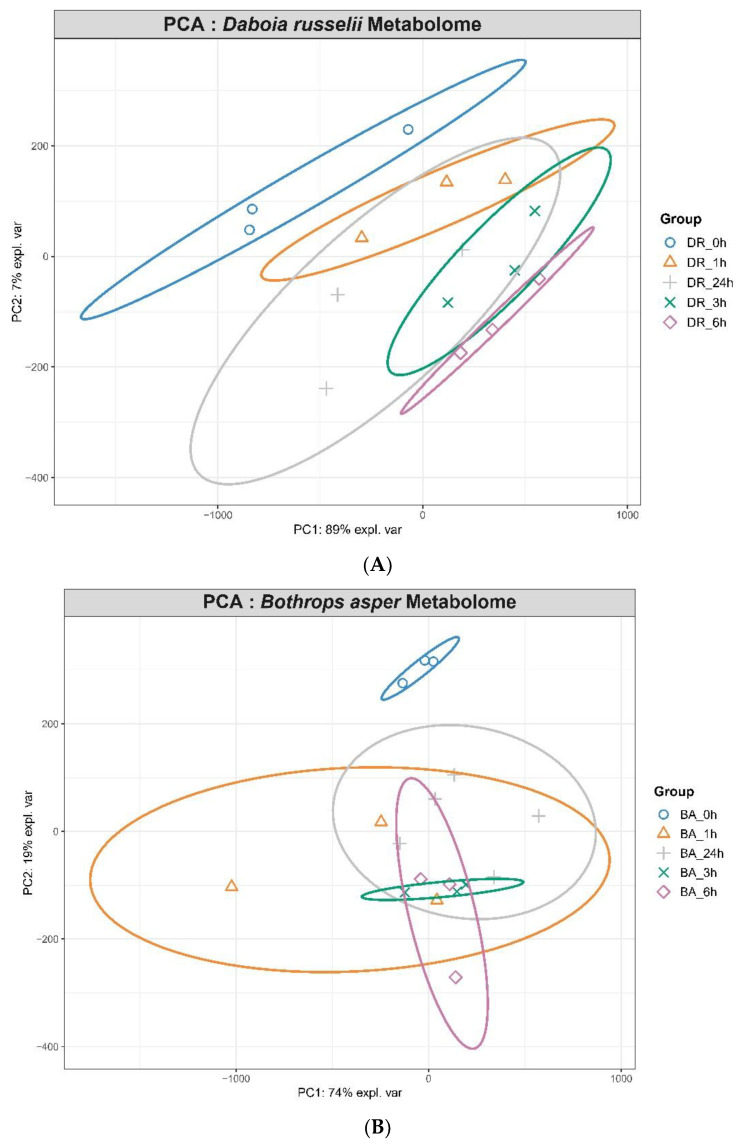

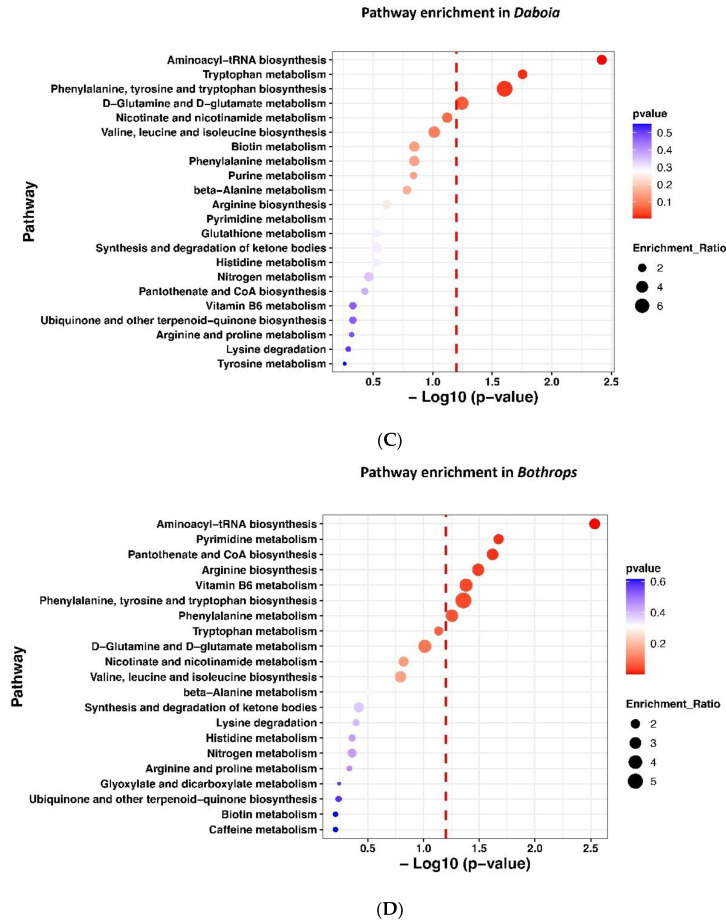
(**A**) PCA plots of metabolome changes in plasma after injection of *D. russelii* (**A**) and *B. asper* (**B**) venoms. (**C**,**D**) Pathway Enrichment plots of dysregulated metabolites in plasma after injection of *D. russelii* (**C**) and *B. asper* (**D**) venoms. Vertical interrupted lines represent *p*-value cutoff ≤ 0.05 for selection of dysregulated pathway.

The molecular signatures of samples obtained 24 h after injection of venoms were more similar to the control, whereas a higher level of perturbation was observed in samples collected at 1, 3 and 6 h post venom injection, thus indicating that the metabolic perturbations were returning to baseline level at 24 h. The PCA plots shown in Figure 2A,B indicate that samples from *D. russelii*-injected mice showed 89% explained variance on PC1 while those from *B. asper*-injected mice showed 74%. PCA score plots show that samples from the same treatments clustered together. A clear separation was observed for both venoms between values in control samples from non-envenomed mice and samples from envenomed mice at the four time intervals, hence evidencing an effect of the venoms on the metabolomic profile of mice at all time intervals evaluated.

We applied linear modelling using limma package to the time series data and after applying a *p*-value ≤ 0.01 identified changes in 122 and 116 metabolites in *B. asper* and *D. russelii*-injected mice, respectively. Once these metabolites were identified, we then proceeded with the MSEA (Metabolite Set Enrichment Analysis) implemented in Metaboanalyst platform. MSEA is a way to identify biologically meaningful patterns of significantly enriched metabolites in quantitative data [20].

As shown in Figure 2C,D, the biosynthetic pathways of several amino acids were perturbed by both venoms along with purine and pyrimidine metabolism. We analyzed the enrichment of the KEGG (Kyoto Encyclopedia of Genes and Genomes) metabolic pathways which showed the highest differences between samples from venom-injected mice and those of non-envenomed mice. The main altered pathways corresponded to aminoacyl-tRNA biosynthesis, tryptophan metabolism, phenylalanine, tyrosine and tryptophan biosynthesis, and D-glutamine and D-glutamate metabolism (in the case of samples from *D. russelii*-injected mice), and to aminoacyl-tRNA biosynthesis, pyrimidine metabolism, pantothenate and CoA biosynthesis, arginine biosynthesis, vitamin B6 metabolism, phenylalanine, tyrosine and tryptophane biosynthesis, phenylalanine metabolism (in the case of samples from *B. asper* -injected mice). The heat map of the top dysregulated metabolites revealed similarities and differences in the metabolite changes induced by these venoms in plasma (Supplemmentary Figure S1).

**Figure 3:**
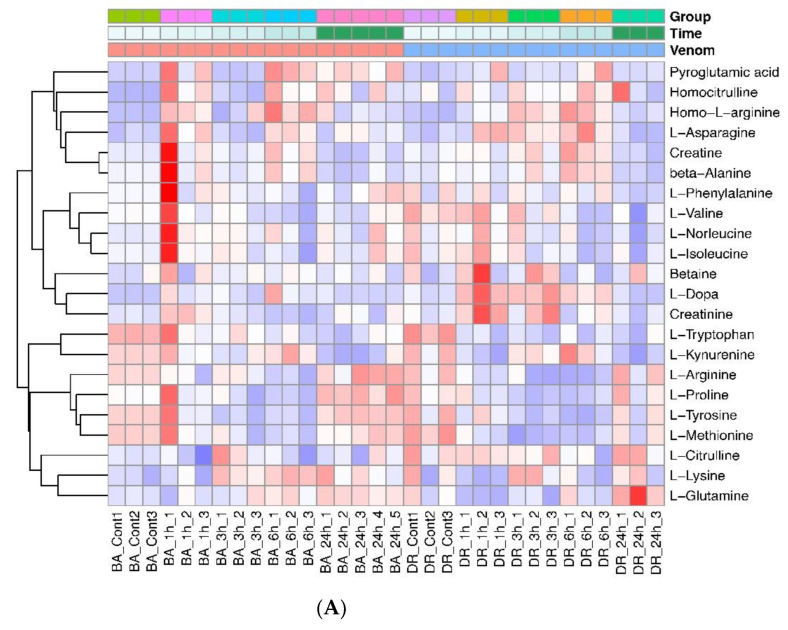

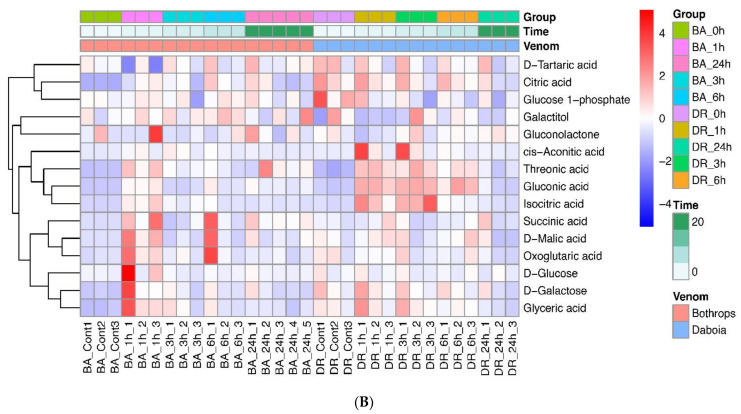
Heatmaps showing the time dependent perturbation of amino acids (**A**) and TCA cycle components and sugars and sugar phosphates (**B**) in the plasma of mice treated with the venoms of *B. asper* and *D. russelii*. Control (cont) corresponds to samples from non-envenomed mice.

**Figure 4:**
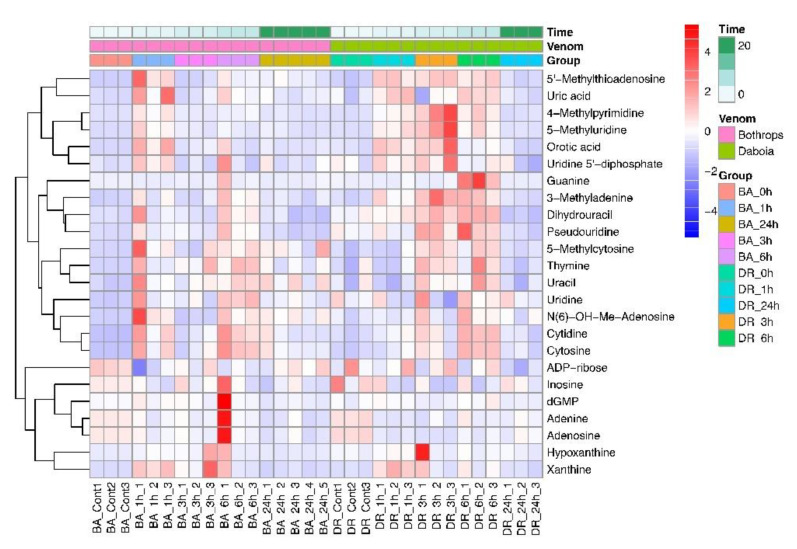
Heatmap showing the time dependent perturbation of purines and pyrimidines in the plasma of mice injected with the venoms of *B. asper* and *D. russelii*. Control (cont) corresponds to samples from non-envenomed mice.

There is a distinct pattern of changes in the level of amino acids as a consequence of venom injection, with evident differences between venoms and time intervals, as shown by the heatmap of Figure 3A. The levels of several amino acids increased in mice injected with both venoms, although those of other amino acids were reduced at some time intervals. Another pathway that was perturbed in envenomed mice is the tricarboxylic acid (TCA) cycle, with increments in some metabolites of this pathway, and there were also changes in the levels of some sugars. Figure 3B shows the heatmap plot of molecules belonging to the tricarboxylic acid (TCA) cycle and sugars from the central carbon metabolism. Regarding purine and pyrimidine metabolism, there was a general trend of increment in several metabolites in samples from mice injected with both venoms, as shown in the heatmap of Figure 4 and in Table 1. In addition, increased levels of cortisol and heme were detected in samples of plasma from envenomed mice, as compared to non-envenomed animals (Table 1).

### 2.2. Lipid Profiling of Plasma from Mice Injected with Venoms

**Figure 5:**
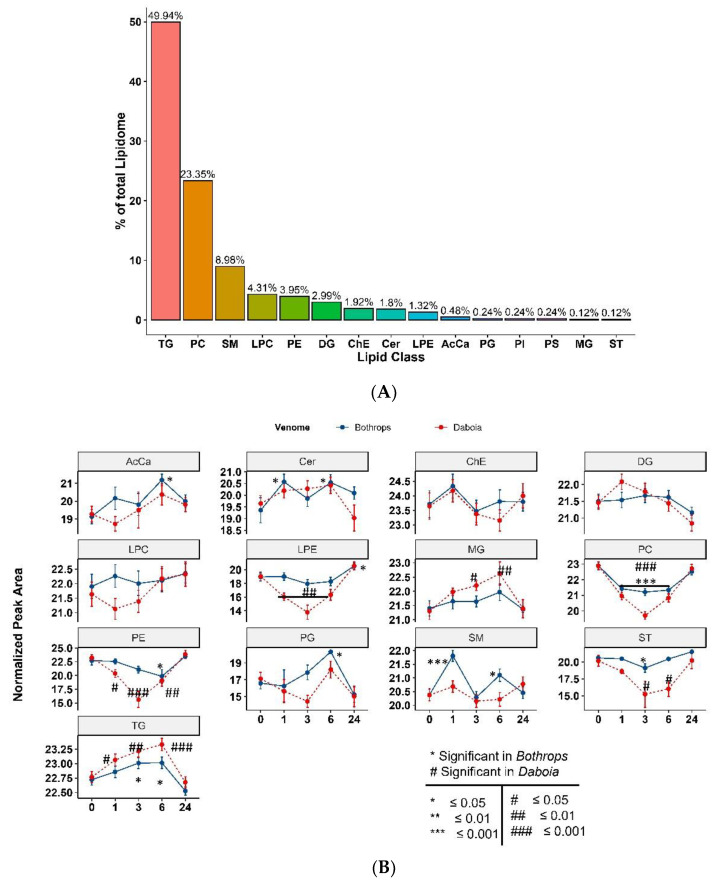
(**A**) Representation of 822 lipid species detected in the lipidomic platform grouped by classes. (**B**) Quantitative comparison of circulating total lipid concentration in plasma samples from mice treated with either *B. asper* or *D. russelii* venoms, grouped by sampling timepoints (*n* = 3). Data on the *y*-axis corresponds to PQN normalized raw average intensity of all lipid species found in a particular group. (Abbreviations: TG: Triaclyglycerol; DG: diacylglycerol; ST: Sterol esters; SM: Sphingomyelin; PG: Phosphatidylglycerol; PE: Phosphatidylethanolamine; PC: Phosphatidylcholine; MG: Monoacylglycerol; LPE: Lysophosphatidylethanolamine; LPC: Lysophophatidylcholine; ChE: Cholesterol ester; Cer: Ceramide; AcCa: Acyl-carnitine). Blue traces: *B. asper* venom; red traces: *D. russelii* venom.

To determine the dynamic changes in the lipids as a consequence of envenoming, we carried out quantitative analysis of plasma lipid levels in plasma samples from mice injected i.m. with 20 µg of either *B. asper* or *D. russelii* venoms, while the control group was not envenomed, as per the workflow depicted in Figure 1. Liquid chromatography mass spectrometry (LC-MS) was used to carry out untargeted lipidomic analysis on the plasma samples. In total, 822 lipids were quantified. In the quality control analysis, the average relative standard deviation (RSD) for the internal standards in the QC samples was 3.94. Lipids were annotated according to the lipid class, acyl-chain detected and level of saturation. For example, PC (16:0/18:2) has a phosphatidylcholine (PC) backbone with two acyl-chains consisting of palmitic acid (16:0) and linoleic acid (18:2). The general trend of class-wise time-dependent changes in samples treated with both venoms is shown in Figure 5A.

Triaclyglyceride (TG; glycerol backbone + 3 acyl chains) was the most abundant lipid class from the entire lipidome panel (49.94%) while PC, SM and LPC were the next most abundant classes (23.35%, 8.98% and 4.31%, respectively) identified in the panel (Figure 5A). Both venoms induced an increment in the levels of TG up to 6 h, returning to lower values at 24 h (Figure 5B). Other classes of lipids that showed significant changes are PC and PE, with a drastic drop in the PC and PE pool up to 6 h, eventually returning to basal values at 24 h (Figure 5B). On the other hand, acyl-carnitines showed an increasing trend after venom treatment, returning to lower levels by 24 h (Figure 5B). Among the several carnitines detected (acetyl-L-carnitine, adipoyl-carnitine, butyryl-carnitine, hexanoyl-L-carnitine, oleoyl-carnitine, palmitoyl-carnitine, propionyl-carnitine, and valeryl-carnitine), a trend of increased levels along time was observed specifically for acetyl-L-carnitine and valeryl-carnitine, while palmitoyl-carnitine decreased (Table 1).

**Figure 6:**
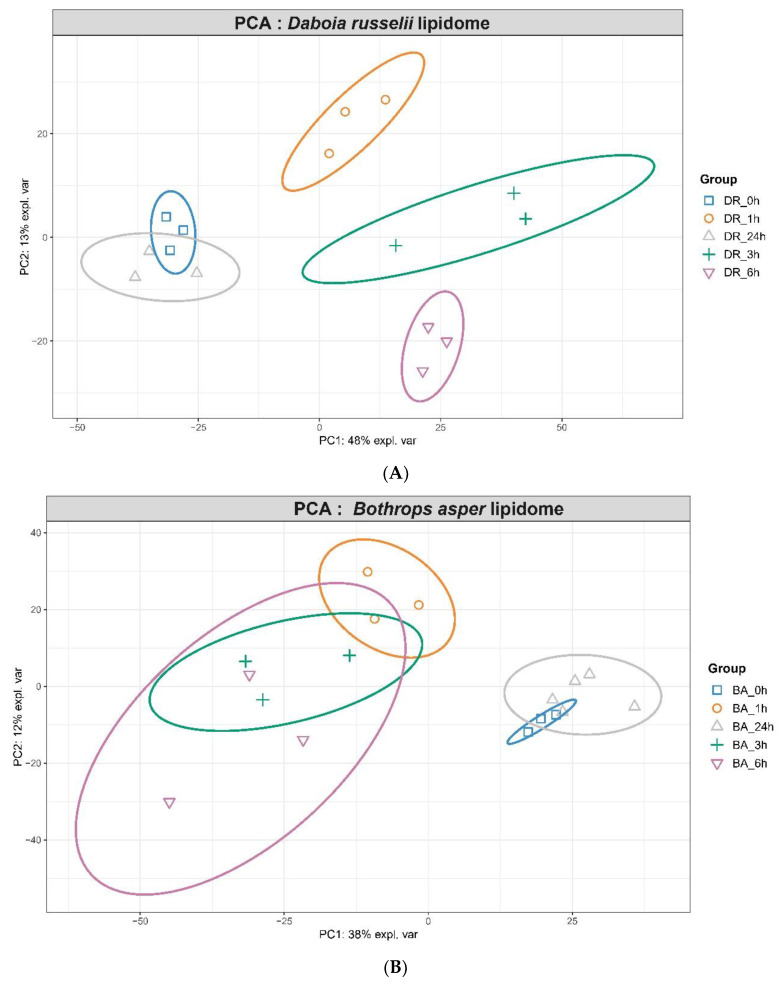

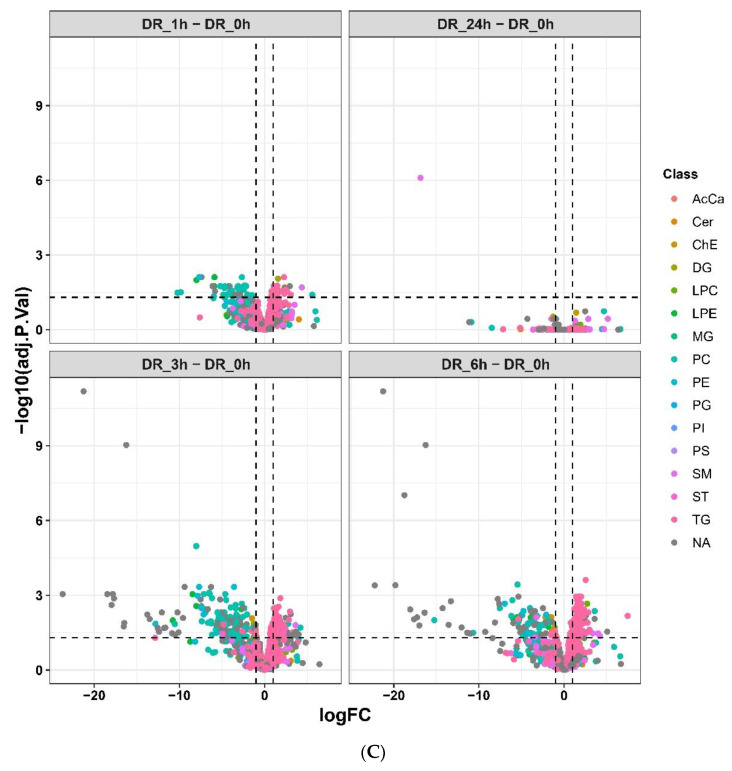

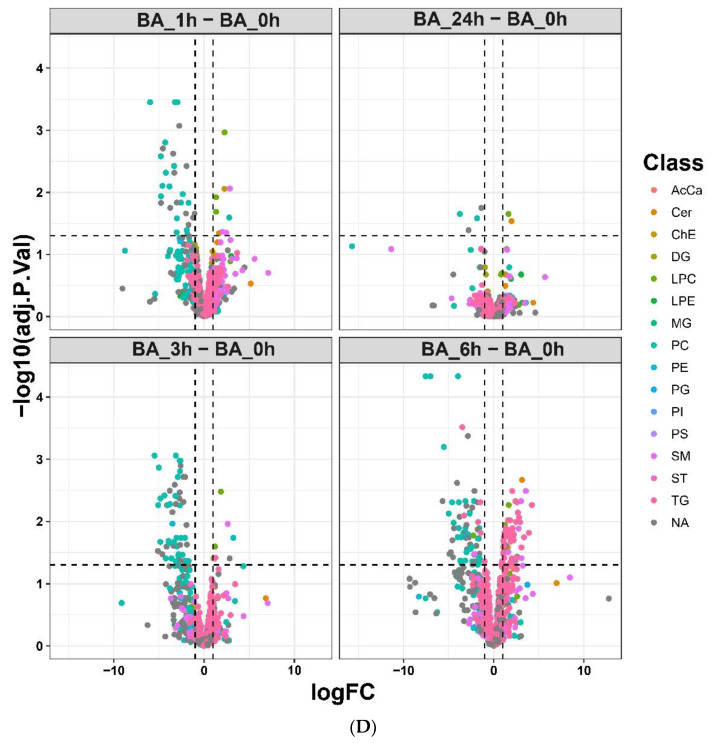
PCA plots of lipidome changes in the plasma of mice injected with *D. russelii* (**A**) and *B. asper* (**B**) venoms. Volcano plots of dysregulated lipids in plasma of mice injected with *D. russelii* (**C**) or *B. asper* (**D**) venoms, with lipid classes color coded.

We also explored the dynamic changes in the global pattern of circulating lipids in the plasma of envenomed mice. The data for multivariate statistical analysis was set as an X-matrix consisting of 32 rows from both *B. asper* and *D. russelii*-injected animals, whereas the Y-matrix represented the 822 metabolites lipid species of various lipid classes. A PCA model was then created to evaluate the distinction of the lipid species level and to identify the relationships between lipidome (matrix X) and changes in the circulating lipid species (matrix Y). Lipids were first analyzed by PCA to visualize the overall distribution of samples. As shown in Figure 6A,B, there is distinct separation between the sampling time points when the data is subjected to unsupervised PCA clustering. For *D. russelii*, there is 48% variance on PC1 while the PC2 showed 13% variance. Similarly, for *B. asper*, PC1 showed 38% variance while PC2 showed 12% variance. In both of these PCA plots, the time 0 samples (obtained from non-envenomed mice) clustered well with the 24 h samples, while other time points are distinctly separated from the control non-envenomed samples. This shows that the maximal variation in the lipidome is captured in samples from 1, 3 and 6 h. To identify the dysregulated lipid species, we then performed pairwise log2fold change calculation comparing every other timepoint with control samples. Finally, to discern highly significant lipid species, we performed one way ANOVA on samples from each treatment to identify lipid species that showed significant changes at *p*-value ≤ 0.01. By using these lipid species from each venom treatment, we created a heatmap of highly dysregulated lipid species (Supplementary Figure S2). We observed that most of the TG levels were increased, especially at 3 and 6 h, and returned to baseline at 24 h. In contrast, phospholipids such as PC and PE showed an opposite trend since they decreased at 1, 3 and 6 h, returning to basal levels at 24 h.

### 2.3. Lipid Ontology Enrichment Analysis

**Figure 7:**
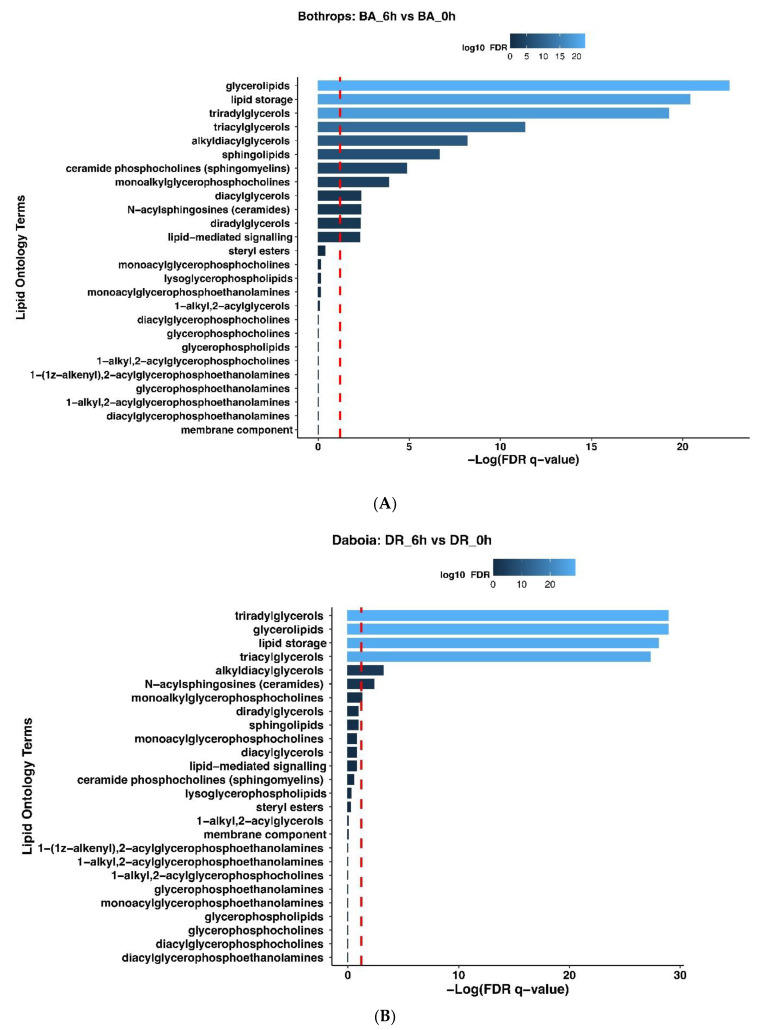
Lipid Ontology Enrichment Analysis: Lipid class overpenetration analysis of plasma collected 6 h after injection from mice treated with *B. asper* (**A**) or *D. russelii* (**B**) venoms. Zero h correspond to samples from non-envenomed mice. Vertical interrupted red lines correspond to −Log_10_ FDR q-value < 0.05.

Univariate analysis comparing the lipid profiles of plasma from mice injected with the venoms identified lipid features with *p*-values below 0.05 after the *t*-test and log2-fold change cut-off, which are represented in the volcano plot (Figure 6C,D). TGs, acyl-carnitines, and ceramides were increased in venoms-treated samples at 1, 3 and 6 h, returning to values similar to those of control samples from non-envenomed mice at 24 h. In contrast, PCs, PE, LPEs, LPCs and STs decreased in envenomed mice when compared to control non-envenomed mice. Lipid ontology (LION) enrichment was performed for lipid class overrepresentation analysis (ORA). Figure 7 shows the ORA analysis for plasma samples collected 6 h after injection from mice treated with venoms, highlighting significant enrichment of terms related to lipid storage, TG, and sphingolipids as compared to control samples. Species such as glycerolipids, triacylglycerols, alkyldiacylglycerols, triradylglycerols, diacylglycerols and monoalkylglycerophosphocholines (i.e., glycerophospholipids containing an alkyl ether substituent) were enriched in samples from envenomed mice (FDR q-value < 0.05). Complete data for annotated metabolites and lipids along with their molecular formula, precursor *m/z*, classification, raw peak intensities, and differential expression analysis is provided as Supplementary Information Tables S1 and S2, respectively.

## 3. Discussion

Viperid snake venoms induce prominent local and systemic pathological and pathophysiological effects which depend on the direct action of toxins on cells, extracellular matrix, and plasma, as well as on indirect effects emerging from the activation of endogenous mechanisms. Venoms elicit strong inflammatory reactions and release DAMPs from affected tissues [5,6], which in turn participate in the complex landscape of physiological alterations. In this work we have explored another aspect of the effects induced by venoms, i.e., the changes in the metabolism, as reflected by metabolomic and lipidomic analyses. Our findings reveal significant perturbations in several metabolic pathways, as an adaptive mechanism to maintain homeostasis, thus contributing to a better understanding of this poorly studied aspect of envenomings.

The venoms used in this study were selected because they have toxicological profiles which show similarities, while also presenting differences in terms of proteomic composition and in the vascular and inflammatory alterations they cause [18,19,21,22,23]. A sublethal dose of 20 µg venom was injected in the gastrocnemius muscle of mice, which has been used in previous studies [19]. A qualitatively similar pattern of metabolomic and lipidomic alterations was observed between venoms, with significant changes in more than 100 metabolites as compared to non-envenomed mice. The main changes were observed in samples collected at 1, 3 and 6 h after envenoming. The PCA analysis of metabolomics revealed an overlap at the four time intervals, which clearly differed from non-envenomed samples. In the case of lipidomics there was an overlap in the samples collected at 24 h with those of non-envenomed mice. This underscores that this envenoming model is characterized by a rapid and transient pattern of metabolic alterations, which then tends to return to normal levels. It would be of interest to study experimental models of envenoming of higher severity, using higher doses of venoms, to assess whether the metabolic changes continue to be altered at 24 h.

Several general trends were observed. Both venoms induced significant changes in amino acids, as well as in several purines and pyrimidines, and in some metabolites of the TCA cycle. Accordingly, KEGG analysis identified metabolic pathways that showed the highest changes, i.e., aminoacyl tRNA synthesis and amino acid biosynthesis and metabolism pathways. Regarding lipid metabolism, there was an increase in TG, ceramides, and several acyl carnitines, concomitantly with a drop in the levels of some phospholipids and lysophospholipids. The lipid ontology enrichment analysis identified a significant enrichment in lipid storage, sphingolipid, glycerolipids and TG metabolites.

Venom injection constitutes an injury and, therefore, it is of interest to relate the metabolic changes observed in this experimental model of envenoming in the light of findings of metabolomic studies in critically injured patients in diverse pathologies. Trauma-dependent metabolic signatures have been associated with lipolysis and fatty acid mobilization, changes in amino acid levels, nucleotide breakdown, increased glycolysis, plasma elevation of TCA intermediates, and accumulation of ketone bodies, among other alterations [24]. Some of these changes were observed in our study, hence suggesting that at least some of the metabolic alterations in envenomed mice are the result of stereotyped reactions of the organism to trauma and injury. In the case of envenoming, however, the changes in some metabolites, such as phospholipids and amino acids, might be also related to the action of venom enzymes, e.g., phospholipases A_2_ (PLA_2_s) and proteinases, on tissue and blood substrates. Nevertheless, the fact that these venoms differ in their proteinase and PLA_2_ activities [25,26,27], while inducing a similar profile of metabolic alterations, suggest that metabolic changes are mainly due to the organism’s response to venom-induced injury. The observed elevated levels of cortisol, a key glucocorticoid hormone related to a response to stress [28], further support the concept that the reaction of the organism to envenoming is associated with a stress response secondary to the pain and tissue damage inflicted by the venoms.

Alterations in the lipidome secondary to envenoming are related to a drop in the levels of phospholipids, especially phosphatidylcholine and phosphatidylethanolamine, and increments in the overall levels of acyl carnitines. This is compatible with increased phospholipid hydrolysis, together with fatty acid mobilization, as reflected by the levels of several acyl carnitines. Such phenomena might depend on the catalytic action of endogenous PLA_2_s [29], added to the action of venom PLA_2_s. In contrast to phospholipids, the levels of TG increased in samples from envenomed mice, hence implying a process of storage of these lipids. This may be related to the formation of lipid droplets in diverse types of cells, as previously shown in macrophages incubated with a PLA_2_ from the venom of *B. asper* [30].

The patterns observed in amino acid metabolism are complex, since the levels of some amino acids increased, while others decreased, in envenomed mice. This might be the consequence of the concomitant action of different processes, such as proteolysis, protein synthesis, and the use of some amino acids for energy production. The net effect of these and other processes is likely to result in a variable pattern depending on the particular amino acid. A previous study of metabolic changes in critically injured patients revealed a general trend to increase in the levels of amino acids [24]. The difference with our findings may be related to the severity of the injury, since our model corresponds to a moderate severity of envenoming in mice while Peltz et al. analyzed samples from severely injured patients [24]. Accordingly, the KEGG analysis indicated that metabolic pathways related to the metabolism and synthesis of some amino acids are among the most altered pathways in envenomed mice. Moreover, aminoacyl-tRNA biosynthesis pathway was significantly altered in the case of both venoms. Coincidentally, in a pig model of envenoming by the elapid snake *Bungarus multicinctus*, one of the pathways showing a high level of alteration was that of aminoacyl-tRNA biosynthesis. In addition, there were significant changes in L-leucine, L-tryptophan, D-proline, glycine, and L-glutamine promoted by this elapid venom [10]. Likewise, changes in some amino acid levels and metabolic pathways were described in people who had suffered stings by honeybees [31], as well as in rats exposed to this venom [11]. In the case of purine and pyrimidine metabolites, a trend of increment was observed, in coincidence with observations in models of traumatic injury [24]. The elevated levels of some intermediates of the TCA cycle are related to the energetic requirements of the organism under stress. Moreover, increases in metabolites of the TCA cycle have been associated with metabolic acidosis [32]. In addition to the increased metabolism of glucose, typical of stress situations, there is also the channeling of lipids and amino acids to fuel the TCA cycle.

Snake venoms contain some of the metabolites detected in this study, such as citrate [33] and purine nucleosides [34]. Moreover, venom 5′ nucleotidase is known to generate adenosine through the hydrolysis of ATP released from cells damaged by toxins [35]. Venoms may directly contribute through these molecules and mechanisms to changes in the metabolome and lipidome. However, it is suggested that the vast majority of the metabolic changes described in this study are the consequence of the response of the organism to venom-induced injury and not to a direct contribution of venom components.

The metabolite heme was increased in the plasma of mice injected by these venoms. This is likely the result of venom-induced intravascular hemolysis whose pathogenesis might be related to the formation of microthrombi, owing to the procoagulant components present in these venoms [36,37,38,39], which in turn may contribute to a thrombotic microangiopathy associated with hemolysis [40]. The heme iron may contribute to the generation of reactive oxygen species, which are known to play a role in tissue alterations in snakebite envenoming [41]. Other detected metabolites which have been related to oxidative stress and to defects in mitochondrial fatty acid beta oxidation are allantoin and isobutyrylglycine, respectively [42,43]. Moreover, glutathione, whose levels are related to changes in the redox status, was also detected in our analysis. This observation calls for further studies on the imbalance in oxidative/antioxidative mechanisms in envenomings.

Although most of the metabolic changes described in this study are likely to reflect stereotypic adaptive responses of the organism to injury and stress, and hence play homeostatic roles, some of these outcomes might also contribute to the pathophysiology of envenoming. The increase in the levels of the group heme constitutes an example, as discussed above. Likewise, phospholipid hydrolysis, by releasing arachidonic acid, contributes to the synthesis of eicosanoids, such as prostaglandins and leukotrienes, which induce a variety of inflammatory actions [6]. In agreement, our study revealed changes in the levels of the eicosanoid 9-hydroxyeicosatetranoic acid (9-HETE). Moreover, niacinamide, which increased in plasma in envenomed mice, has been related to the breakdown of NAD in traumatic brain injury and with post-traumatic motor and cognitive sequelae [24]. It would be relevant to assess whether some of the transient mental confusion effects described in some patients suffering snakebite envenomings might be due to the action of some of these metabolites in the brain. Another subject that requires investigation is the relationship between the plethora of inflammatory mediators released by these venoms [19,44] and the dysregulation of the metabolic pathways responsible for the changes in the metabolome and the lipidome. Furthermore, possible changes in the permeability of the intestinal barrier in the course of viperid snakebite envenoming, due to local ischemia, may contribute with gut microbiome metabolites, an intriguing possibility deserved to be investigated. These possibilities underscore the multifactorial causality of metabolic alterations in envenoming.

In conclusion, the venoms of *B. asper* and *D. russelii* induce a complex transient pattern of alterations in the concentrations of metabolites and lipids in the plasma of mice. These changes have features in common with the typical reaction of the organism to traumatic injuries. The role that these alterations play in the overall pathophysiology of envenoming remain to be studied, and the information provided in this investigation constitutes a valuable body of data upon which new hypotheses can be generated to further expand our understanding of the complexity of snakebite envenoming, as well as to find novel therapeutic and diagnostic tools to improve the management of this neglected tropical disease.

## 4. Materials and Methods

### 4.1. Venoms

*Bothrops asper* venom is a pool obtained from more than 40 adult specimens collected in the Pacific region of Costa Rica and kept at the serpentarium of Instituto Clodomiro Picado (Universidad de Costa Rica, San José, Costa Rica). After collection, venom was pooled, lyophilized, and stored at −20 °C. *Daboia russelii* venom comes from specimens collected in Pakistan, and was purchased from LATOXAN (Portes-les-Valence, France; code L1132A; Lot: 015.051). It was also stored at −20 °C.

### 4.2. Experimental Model of Envenoming in Mice

Groups of four mice (CD-1 strain, 18–20 g body weight) received an intramuscular injection, in the right gastrocnemius muscle, of 20 µg of either *B. asper* or *D. russelii* venom, dissolved in 50 µL of 0.12 M NaCl, 0.04 M phosphates, pH 7.2 solution (PBS). This dose was selected because it has been used in previous comparative studies with these two venoms [18,19]. A control group of non-envenomed mice was included. At various time intervals after injection of venom or in the group of non-envenomed mice, a blood sample was collected from the ocular venous plexus under isofluorane anesthesia using 3.8% sodium citrate as anticoagulant in a 1:10 proportion (anticoagulant:blood). After centrifugation at 1300× *g*, plasma was collected and freeze-dried. Experiments involving the use of mice were approved by the Institutional Committee for the Care and Use of Laboratory Animals (CICUA) of the University of Costa Rica (permission CICUA-025-15) and meet the International Guiding Principles for Biomedical Research Involving Animals (CIOMS).

### 4.3. Extraction of Hydrophilic and Hydrophobic Metabolites

Plasma from mice injected with venoms or PBS was resuspended in 200 µL of water and 50 µL of plasma was used for extraction of metabolites and lipids. To each tube, 600 µL of −20 °C cold chloroform:methanol (1:2) mixture was added. Tubes were vortexed and shaken vigorously for 30 min at 4 °C in a temperature controlled thermal mixer (Thermo Scientific, Waltham, MA, USA). Four hundred microliters water were added to induce phase separation. The top aqueous methanolic phase was recovered as metabolite mixture. A second extraction was performed by adding 500 µL of 80% methanol. Top aqueous methanolic phase was recovered and combined with the previous extract, while the bottom part was collected for lipidomic analysis. Metabolite mixtures were stored in Eppendorf tubes at −80 °C.

For lipid extraction, additional 500 µL of chloroform along with 400 µL of water was added for phase separation. The lower chloroform phase was recovered as lipid mixture in 2 mL screw cap glass vials. Lipids were dried under gentle stream of N_2_ at 45 °C using Reacti-Vap™ Evaporators (Thermo Scientific, Waltham, MA, USA). Dried lipids were stored in −80 °C until analysis. Before drying, 10 µL of Avanti Splash Lipidomix II (Avanti Polar Lipids, Birmingham, AL, USA) was added to each tube.

### 4.4. UPLC-MS/MS Analysis and Identification of Metabolites

Each extract was dried overnight in a speedVac and resuspended in 100 µL of 0.1% formic acid. A pooled QC sample was created by aliquoting 10 µL of sample from each tube and 2 µL were injected per sample per ionization mode (38 × 2 = 76 injections + 3 × 2 blanks + 6 × 2 QC samples). Pooled QC sample was injected at the beginning and end of the MS sequence run and additional QC samples were injected after every 10 sample injections for continued assessment of chromatography quality.

Samples were analyzed in untargeted fashion by LC-HRMS. Samples were injected randomly via a Thermo Vanquish UHPLC and separation of the polar metabolites was achieved using Waters BEH C18 column (100 mm × 2.1 mm, 1.9 µm) operated at 30 °C and at flow rate of 250 µL/min. The injection volume was 2 µL. For the 36 min gradient, the standard mobile phase for RPLC was A = 0.1% formic acid in water and B = 0.1% formic acid in methanol. The linear elution gradient was as follows: 0–2.0 min at 5% B, 2.0–25.0 min at 50% B, 25.1 to 32.0 min at 95% B and, finally, 32.1 to 36 min revert to 5% B to re-equilibration for next injection. Spectra were acquired on Thermo IDX™ Tribrid MS (Thermo Scientific, Waltham, MA, USA) using both positive and negative ionization modes. The heated electrospray ionization (HESI) source was operated at 3.5 kV and 2.5 kV for positive and negative modes, respectively. Ion source sheath gas was set at 35 and auxiliary gas at 7. Ion transfer tube temperature was maintained at 275 °C while vaporizer temperature was maintained at 320 °C. The instrument was set to acquire over the *m/z* range 67–1500, in full MS mode (1 µscan) at a resolution of 120,000 at a normalized AGC Target of 25% and 50 milliseconds of maximum injection time was allowed. RF lens amplitude was set at 35%. Tandem MS/MS was carried out by applying quadrupole isolation with an isolation window of 1.6 *m/z*. Activation type was set at HCD and masses were fragmented with HCD Assisted Collision Energy (%) of 25, 30, 35. Three blank samples were included in the analysis to identify background ions and to remove them subsequently. Prior to running the samples, the instrument was calibrated using Pierce FlexMix solution. Before running the actual experiment, LC-MS system was stabilized by running 3–4 wash runs followed by 4 blank runs to ensure stable background signal, and lastly a commercial amino acid mixture (Promega) was run as part of system stability analysis to ensure the column chromatograph was in good condition.

Once the data were acquired, samples were analyzed using the open-source software MS-DIAL [45,46,47]. Data acquired using full scan MS1 used for quantification and data dependent acquisition (DDA) mode were used for spectral identification of the metabolites. The MS1 tolerance was set to 0.01 Da and the MS2 set to 0.025 Da. For peak picking mass slice width was set to 0.1. For peak alignment, the maximum retention time tolerance was set at 0.2 min, MS1 tolerance was set to 0.015. Peaks were identified by searching the MS2 spectra in MS-DIAL public database (290,915 records (14 August 2020) for positive and 36,848 entries for negative mode) using a mass tolerance of 0.01 Da for MS1 and 0.05 Da for MSMS with identification score cutoff of 70. Additionally, peaks were also searched against in-house IORA library (in both positive and negative mode) with a mass tolerance of 0.01 and identification cutoff of 90%.

Raw data from MS-DIAL was exported out as txt file and further processing was done using R. The first blank injection signal subtraction, and features that have RSD > 30% in the QC sample were discarded. Further, data from both negative and positive polarity were combined, and again examined for duplicates and subsequently cleaned. The final data matrix thus obtained contains 63 negative mode metabolite and 317 positive mode metabolites. All metabolite identifications met MSI Level 1 and 2 requirements [48].

### 4.5. UPLC-MS/MS Analysis and Identification of Lipids

The dried lipid extract was reconstituted in 110 µL of methanol:isopropanol (1:1). Approximately 100 µL was recovered and 10 µL was aliquoted to generate a pooled QC sample. Samples were transferred to borosilicate glass inserts kept inside a screw-capped glass autosampler vials (Agilent, Santa Clara, CA, USA). All the samples were run in a randomized fashion in both positive and negative ionization modes. MS data was acquired on Thermo Orbitrap IDX MS connected to Vanquish UPLC system. Lipid extract was separated using Ascentis^®^ Express C18 (Sigma-Aldrich, St Louis, MO, USA, 2.1 mm × 100 mm, 2.7 µm) operated at 55 °C and a flow rate of 260 µL/min. Mobile phase A was 60:40 acetonitrile/water and mobile phase B was 90:10 isopropyl alcohol/acetonitrile; both A and B contained 10 mM ammonium formate and 0.1% formic acid. Linear gradient was started at 32% B at 0 min and ramped to 45% B at 4 min. Gradient ramped to 75% B at 18 min and further increased to 95% at 25 min. From 25.1 to 33.0 min the buffer B was reduced to 32% for re-equilibration and preparation for the next injection. Data was acquired in full scan mode at 250–1500 at a resolution of 120,000 with a scan range of 1.5 sec. DDA MS2 scans were obtained with an Orbitrap resolution of 15,000 and stepped collision HCD energy of 25,30,35 was used.

Raw data files were searched in LipidSearch software V4.1 for lipid identification. Search type was set to Product Search. Both precursor and product tolerance were set to 5 ppm with a relative intensity cut-off of 1%. For adducts, negative mode adduct set as M-H and M + HCOO while for positive mode adduct was set as M + H and M + NH_4_. Peak integration was performed by baseline subtraction, peak smoothing, and deconvolution. Finally, peaks were aligned using alignment module with a RT tolerance of 0.25 min. For calculating group peak area max integration algorithm was used. After the search and alignment, peak data was exported out as txt file, brought into R and further analysis was performed in R.

To reduce misidentification, each identified lipid was manually investigated to validate the assignments. Following validation considerations were taken: (1) Positive and negative MS/MS spectra match the expected fragments, (2) The retention time is compatible with the lipid class identified and (3) the peak shape is acceptable, (4) LipidSearch grades the identification into categories: Grade A: Class and all FA chains are completely identified. Grade B: Both class specific ions and FA derived product ions are detected. Grade C: Either class specific ion or FA derived product ions are detected. Grade D: unable to identify lipid structure. For example: PC (16:0/18:1) can be graded as Grade A and B, since it identifies molecular species with specific fatty acid chains. Another example could be TG (64:10), here we identified class specific ions but no FA derived product ions hence graded as Grade C while TG (20:2/22:5/22:5) could be classified as Grade A and B, since both class specific ions and product ions were identified. We removed spurious identification based on this classification and retained only grade A, B and C annotations. Duplicated peaks were manually curated and lipid species that shows highest intensity was retained. For data analysis of the lipid species, a combined datamatrix of both neg and positive data was generated. Probabilistic Quotient Normalization (PQN) [49] was applied before performing discovery of dysregulated lipids.

### 4.6. Statistical Analysis of Metabolomics and Lipidomics Experiments

Raw peak intensities were analyzed in R version 4.2.0. Metabolite peak intensities were first median normalized and autoscaled. For multivariate analysis, the R package mixOmics [50] was used to analyze venom-induced changes over time. For the discovery of dysregulated metabolites, linear model with covariate adjustment analysis was performed based on limma R package [51]. Changes in metabolites that have adjusted *p*-value ≤ 0.05 were deemed significant. Metabolite enrichment analysis was conducted in MetaboAnalyst v5.0 [52,53] using KEGG pathways overrepresentation analysis (ORA; hypergeometric test).

For lipidomics data analysis, peak areas exported out as txt file from LipidSearch v4.0 and brought into R and analyzed using R package lipidr [54]. Probabilistic Quotient Normalization (PQN) [49] was applied before performing DE analysis of the lipid species. For multivariate analysis, R package mixOmics was used [50]. For DE analysis, using samples at 0 h as reference, log_2_fold change and *p*-value was computed as a pair-wise analysis. Further lipid ontology analysis was performed using online tool LION [55] to identify enriched terms related to lipid metabolism after treatment with snake venoms.

## Figures and Tables

**Table 1:** Metabolites in plasma samples from mice injected with the venoms of *D. russelii* or *B. asper* which show the highest difference when compared to samples from control, non-envenomed mice. Using R limma package, linear modelling of the time series data was performed assuming samples from non-envenomed mice as a reference group. The list provided in the table is filtered based on the adjusted *p*-value ≤ 0.01.

Metabolite	KEGG	Ion Mode	*Bothrops asper*						*Daboia russelii*					
			1 h	3 h	6 h	24 h	*p* Value	adj. *p* Value	1 h	3 h	6 h	24 h	*p* Value	adj. *p* Value
**Amino Acids**														
L-Tryptophan	C00078	ESI+	−1.90	−1.60	−2.29	−2.66	0.00006970	0.000883	−2.33	−2.45	−2.27	−2.04	0.0001920	0.004763
L-Methionine	C00073	ESI+	−1.98	−1.97	−2.63	−1.36	0.00027800	0.001623	−1.86	−2.56	−2.30	−0.95	0.0000760	0.004763
L-Proline	C00148	ESI+	−0.72	−1.13	−1.64	0.87	0.00038100	0.002022	−1.11	−1.93	−1.83	0.10	0.0010000	0.006088
L-Tyrosine	C00082	ESI+	−1.74	−2.20	−2.31	−0.52	0.00004150	0.000679	−1.21	−1.99	−2.28	−0.44	0.0008980	0.005881
L-Valine	C00183	ESI+	−1.81	−1.14	−2.72	−1.71	0.00020600	0.001423	−0.97	−1.56	−2.59	−1.34	0.0021790	0.009202
N-Isobutyrylglycine		ESI−	1.99	0.58	2.08	0.76	0.00315100	0.008740	1.41	2.23	2.05	0.34	0.0004520	0.004911
**Carnitines**														
Adipoyl-carnitine		ESI+	0.08	0.29	1.80	−0.13	0.03391800	0.054383	−0.10	0.79	2.09	−0.14	0.0016980	0.008064
L-Acetylcarnitine	C02571	ESI+	1.88	1.00	2.40	0.75	0.00134300	0.004858	1.10	2.17	1.75	0.20	0.0018370	0.008345
Oleoyl-carnitine		ESI+	0.15	−1.28	0.07	−1.36	0.04880200	0.072441	−0.25	0.07	−1.52	−1.75	0.0081700	0.021035
Propionylcarnitine	C03017	ESI+	0.87	1.04	−0.46	0.40	0.23318000	0.274330	−0.51	−1.24	−0.47	1.11	0.0101400	0.023784
Valeryl-carnitine		ESI+	1.42	0.16	2.45	1.35	0.00014700	0.001295	−0.09	2.04	1.70	0.54	0.0007960	0.005502
L-Carnitine	C00318	ESI+	−1.89	0.45	−1.57	−0.95	0.00020300	0.001423	−0.33	−0.96	−0.85	1.21	0.0083800	0.021230
Butyryl carnitine	C02862	ESI+	0.67	0.55	−0.27	0.63	0.66292000	0.677180	−1.55	−2.05	−0.37	−0.44	0.0087620	0.021762
Deoxycarnitine	C01181	ESI+	−2.44	−0.46	−1.26	−1.38	0.00174500	0.005785	−0.41	−1.45	−1.55	0.09	0.0350600	0.064361
Palmitoylcarnitine	C02990	ESI+	−1.23	−2.05	−0.98	−2.18	0.00640900	0.014670	−1.48	−1.36	−2.22	−2.66	0.0002780	0.004763
**Indoles**														
5-Hydroxyindoleacetic acid	C05635	ESI+	−1.85	−1.61	−0.92	−2.64	0.00022700	0.001488	−2.26	−1.04	−0.65	−2.01	0.0019850	0.008668
1H-Indole-3-carboxaldehyde	C08493	ESI+	−2.11	−1.78	−2.36	−2.64	0.00002820	0.000649	−2.31	−2.41	−2.20	−1.87	0.0004140	0.004763
2-Methylindole		ESI+	−2.03	−1.75	−2.37	−2.64	0.00003750	0.000649	−2.33	−2.45	−2.25	−1.99	0.0002320	0.004763
3-Indoleacrylic acid		ESI+	−2.01	−1.74	−2.34	−2.67	0.00003480	0.000649	−2.34	−2.45	−2.24	−1.97	0.0002290	0.004763
**Lipids**														
Glycerophosphocholine	C00670	ESI+	−2.44	−0.85	−2.30	−1.45	0.00009900	0.001140	−0.60	−1.42	−1.64	0.83	0.0002620	0.004763
1-heptadecanoyl-2-hydroxy-sn-glycero-3-phosphocholine		ESI+	−1.11	−1.90	−2.25	−2.48	0.00014500	0.001295	−0.58	−1.69	−2.39	−1.93	0.0005410	0.005174
1-pentadecanoyl-2-hydroxy-sn-glycero-3-phosphocholine		ESI+	0.55	−0.93	−1.57	−1.62	0.00080500	0.003517	0.64	−1.02	−1.76	−1.44	0.0003930	0.004763
1-Stearoyl-sn-glycero-3-phosphocholine		ESI+	−0.78	−1.77	−2.01	−2.24	0.00147800	0.005250	−0.45	−1.64	−2.37	−1.76	0.0006960	0.005502
LPC 16:0		ESI+	−1.23	−1.82	−2.15	−2.40	0.00099700	0.003986	−0.26	−1.52	−2.46	−1.86	0.0000756	0.004763
LPC 18:1		ESI+	−1.59	−2.38	−2.61	−1.99	0.00004890	0.000688	−1.11	−2.29	−2.48	−1.90	0.0002810	0.004763
LPC 18:2		ESI+	−2.27	−2.35	−2.41	−1.00	0.00000598	0.000649	−2.10	−2.42	−2.39	−1.16	0.0001370	0.004763
10-hydroxy capric acid	C02774	ESI−	0.41	−0.57	0.01	2.13	0.00006140	0.000804	−1.72	−1.99	−1.39	−0.01	0.0015520	0.007560
**Nucleic acid**														
3-Methyladenine	C00913	ESI+	1.00	0.47	2.43	0.74	0.00285600	0.008329	0.98	2.35	1.55	0.19	0.0009950	0.006088
4-Methylpyrimidine		ESI+	2.40	0.48	1.10	0.21	0.00072200	0.003226	0.62	2.33	0.88	0.04	0.0018150	0.008345
5-Methylcytosine	C02385	ESI+	−2.22	−1.03	−2.21	−0.55	0.00026500	0.001599	−1.35	−1.77	−1.99	0.25	0.0001610	0.004763
5’-Methylthioadenosine	C00170	ESI+	2.43	0.12	1.16	1.19	0.00038900	0.002024	2.17	2.18	1.73	0.51	0.0004070	0.004763
5-Methyluridine		ESI+	2.80	0.94	1.01	1.10	0.00011300	0.001197	0.84	2.36	0.76	0.10	0.0020250	0.008746
**Organic acid**														
Benzoate	C00180	ESI+	−1.98	−2.24	−2.39	−0.78	0.00003310	0.000649	−1.14	−1.90	−2.18	−0.22	0.0010090	0.006088
**Others**														
2-Amino-6-methoxybenzothiazole		ESI+	1.77	0.37	2.24	1.14	0.00188800	0.005928	1.31	2.36	1.60	0.09	0.0003030	0.004763
Allantoin	C01551	ESI−	1.40	0.61	2.51	1.10	0.00159000	0.005596	1.21	2.39	1.62	0.15	0.0003650	0.004763
2-Pyrrolidinone	C11118	ESI+	−2.29	−2.03	−2.40	−2.16	0.00020000	0.001423	−0.93	−2.26	−2.36	−1.50	0.0007390	0.005502
1,5-Naphthalenediamine	C19463	ESI+	−2.07	−1.71	−2.33	−2.66	0.00003420	0.000649	−2.33	−2.44	−2.21	−1.94	0.0002910	0.004763
2-Aminonaphthalene	C02227	ESI+	−2.08	−1.76	−2.35	−2.65	0.00003180	0.000649	−2.32	−2.43	−2.21	−1.95	0.0003220	0.004763
H-Pro-Hyp-OH		ESI+	2.03	0.28	1.86	1.43	0.00253000	0.007512	2.21	2.09	1.64	0.41	0.0003790	0.004763
9,10-Phenanthrenedione	C03243	ESI+	−0.83	−0.98	0.75	−1.91	0.00013700	0.001295	−0.63	0.36	1.33	−1.32	0.0004260	0.004763
2-Phenylacetamide	C02505	ESI+	−1.94	−2.27	−2.37	−0.75	0.00003260	0.000649	−1.16	−1.92	−2.20	−0.31	0.0011470	0.006602
Diphenylphosphine oxide		ESI+	−2.11	−2.33	−2.23	−1.49	0.00068500	0.003099	−1.58	−2.43	−2.51	−0.94	0.0000720	0.004763
Piperidine	C01746	ESI+	−1.83	−1.14	−2.75	−1.50	0.00017500	0.001415	−0.44	−1.60	−2.27	−0.48	0.0021420	0.009144
2,3-Butanediol	C00265	ESI+	−1.99	−2.25	−2.37	−0.77	0.00003170	0.000649	−1.15	−1.89	−2.22	−0.27	0.0009980	0.006088
Niacinamide	C00153	ESI+	1.59	2.28	0.39	0.10	0.00013400	0.001295	1.63	2.35	1.19	0.07	0.0002840	0.004763
Methylimidazoleacetic acid	C05828	ESI+	2.55	0.59	1.27	0.42	0.00024800	0.001519	0.63	2.38	0.90	0.09	0.0013150	0.007252
Gluconic acid	C00257	ESI−	2.08	0.57	1.65	2.04	0.00180800	0.005815	2.10	2.11	1.77	0.37	0.0003590	0.004763
Homatropine		ESI+	1.88	0.02	1.97	1.06	0.00095100	0.003844	1.98	2.64	1.91	1.05	0.0002600	0.004763
2-Aminoacetophenone		ESI+	−1.86	−1.65	−0.95	−2.65	0.00022500	0.001488	−2.32	−1.05	−0.66	−1.89	0.0025300	0.009911
4-Hydroxyquinoline	C06343	ESI−	−0.92	−1.01	−2.24	−2.48	0.00008360	0.000993	0.52	1.63	−0.30	−0.99	0.0007800	0.005502
3-(2-hydroxyphenyl)prop-2-enoic acid	C01772	ESI+	−1.88	−2.25	−2.35	−0.67	0.00003390	0.000649	−1.17	−1.93	−2.21	−0.32	0.0010990	0.006528
6-Quinolinol		ESI+	−1.96	−1.59	−0.94	−2.62	0.00021600	0.001467	−2.34	−1.05	−0.73	−1.98	0.0017590	0.008254
2-aminophenol	C02009	ESI+	−1.95	−2.28	−2.36	−0.78	0.00003510	0.000649	−1.14	−1.89	−2.17	−0.26	0.0013450	0.007300
Cortisol	C00735	ESI+	2.23	0.61	0.41	−0.26	0.00061300	0.002878	1.77	1.06	1.64	−0.46	0.0005450	0.005174
1-Aminocy clopropanecarboxylic acid	C01234	ESI+	−2.31	−2.34	−2.46	−1.49	0.00004570	0.000679	−1.86	−2.45	−2.31	−0.94	0.0001640	0.004763
Heme	C00032	ESI+	2.13	1.64	0.14	0.15	0.00024100	0.001519	2.49	1.25	0.16	0.21	0.0000740	0.004763

## Data Availability

All original mass spectrometry data related to the metabolomics and lipidomics experiment is archived in the online repository (https://www.ebi.ac.uk/metabolights/MTBLS5430/descriptors).

## Supplementary Materials

The following supporting information can be downloaded at: https://www.mdpi.com/article/10.3390/toxins14100657/s1, Table S1: Complete data for annotated metabolites along with their molecular formula, precursor *m*/*z*, classification, raw peak intensities, and differential expression analysis. Table S2: Complete data for annotated lipids along with their molecular formula, precursor *m*/*z*, classification, raw peak intensities, and differential expression analysis. Figure S1: Heatmap of top dysregulated metabolites in plasma samples from mice injected with *B. asper* and *D. russelii* venoms. Control (cont) corresponds to samples from non-envenomed mice. Figure S2: Heatmap of top 80 dysregulated lipids in plasma samples from mice injected with *B. asper* and *D. russelii* venoms. Control (cont) corresponds to samples from non-envenomed mice.

## References

[1] Gutiérrez J.M., Calvete J.J., Habib A.G., Harrison R.A., Williams D.J., Warrell D.A. (2017). Snakebite envenoming. Nat. Rev. Dis. Prim..

[2] Calvete J.J. (2017). Venomics: Integrative venom proteomics and beyond. Biochem. J..

[3] Tasoulis T., Isbister G.K. (2017). A review and database of snake venom proteomes. Toxins.

[4] Warrell D.A. (2010). Snake bite. Lancet.

[5] Rucavado A., Nicolau C.A., Escalante T., Kim J., Herrera C., Gutiérrez J.M., Fox J.W. (2016). Viperid envenomation wound exudate contributes to increased vascular permeability via a DAMPs/TLR-4 mediated pathway. Toxins.

[6] Teixeira C., Moreira V., Gutiérrez J.M., Cavaillon J.M., Singer M.E. (2018). Venoms. Inflammation: From Molecular and Cellular Mechanisms to the Clinic.

[7] Bickler P.E. (2020). Amplification of Snake Venom Toxicity by Endogenous Signaling Pathways. Toxins.

[8] Johnson C.H., Ivanisevic J., Siuzdak G. (2016). Metabolomics: Beyond biomarkers and towards mechanisms. Nat. Rev. Mol. Cell Biol..

[9] Wishart D.S. (2019). Metabolomics for Investigating Physiological and Pathophysiological Processes. Physiol. Rev..

[10] Huang Z., Zhang M., He D., Song P., Mo C., Cheng X., Song T., Li Y., Zhang X., Liao M. (2021). Serum metabolomics of Bama miniature pigs bitten by Bungarus multicinctus. Toxicol. Lett..

[11] Zhao Y., Zhang J., Chen Y., Li Z., Nie H., Peng W., Su S. (2018). Altered Serum Metabolite Profiling and Relevant Pathway Analysis in Rats Stimulated by Honeybee Venom: New Insight into Allergy to Honeybee Venom. J. Agric. Food Chem..

[12] Arjmand M., Akbari Z., Taghizadeh N., Shahbazzadeh D., Zamani Z. (2015). NMR-based metabonomics survey in rats envenomed by Hemiscorpius lepturus venom. Toxicon.

[13] Yuan H., Gao Z., Chen G., Peng C., Sun Y., Jiang B., Zhou H., Cheng Y., Hu F., Zhang Q. (2022). An integrative proteomics metabolomics based strategy reveals the mechanisms involved in wasp sting induced acute kidney injury. Toxicon.

[14] Gutiérrez J.M., Rucavado A., Chaves F., Díaz C., Escalante T. (2009). Experimental pathology of local tissue damage induced by *Bothrops asper* snake venom. Toxicon.

[15] Gutiérrez J.M., Escalante T., Rucavado A. (2009). Experimental pathophysiology of systemic alterations induced by *Bothrops asper* snake venom. Toxicon.

[16] Otero-Patiño R. (2009). Epidemiological, clinical and therapeutic aspects of *Bothrops asper* bites. Toxicon.

[17] Warrell D.A. (1995). Clinical toxicology of snakebite in Africa and the Middle East/Arabian peninsula. Handbook of Clinical Toxicology of Animal Venoms and Poisons.

[18] Rucavado A., Escalante T., Camacho E., Gutiérrez J.M., Fox J.W. (2018). Systemic vascular leakage induced in mice by Russell’s viper venom from Pakistan. Sci. Rep..

[19] Rucavado A., Escalante T., Kalogeropoulos K., Camacho E., Gutiérrez J.M., Fox J.W. (2020). Analysis of wound exudates reveals differences in the patterns of tissue damage and inflammation induced by the venoms of *Daboia russelii* and *Bothrops asper* in mice. Toxicon.

[20] Subramanian A., Tamayo P., Mootha V.K., Mukherjee S., Ebert B.L., Gillette M.A., Paulovich A., Pomeroy S.L., Golub T.R., Lander E.S. (2005). Gene set enrichment analysis: A knowledge-based approach for interpreting genome-wide expression profiles. Proc. Natl. Acad. Sci. USA.

[21] Alape-Girón A., Sanz L., Escolano J., Flores-Díaz M., Madrigal M., Sasa M., Calvete J.J. (2008). Snake venomics of the lancehead pitviper *Bothrops asper*: Geographic, individual, and ontogenetic variations. J. Proteome Res..

[22] Kalita B., Mackessy S.P., Mukherjee A.K. (2018). Proteomic analysis reveals geographic variation in venom composition of Russell’s Viper in the Indian subcontinent: Implications for clinical manifestations post-envenomation and antivenom treatment. Expert Rev. Proteom..

[23] Pla D., Sanz L., Quesada-Bernat S., Villalta M., Baal J., Chowdhury M.A.W., León G., Gutiérrez J.M., Kuch U., Calvete J.J. (2019). Phylovenomics of *Daboia russelii* across the Indian subcontinent. Bioactivities and comparative in vivo neutralization and in vitro third-generation antivenomics of antivenoms against venoms from India, Bangladesh and Sri Lanka. J. Proteom..

[24] Peltz E.D., D’Alessandro A., Moore E.E., Chin T., Silliman C.C., Sauaia A., Hansen K.C., Banerjee A. (2015). Pathologic metabolism: An exploratory study of the plasma metabolome of critical injury. J. Trauma Acute Care Surg..

[25] Gutiérrez J., Gené J.A., Rojas G., Cerdas L. (1985). Neutralization of proteolytic and hemorrhagic activities of Costa Rican snake venoms by a polyvalent antivenom. Toxicon.

[26] Saravia P., Rojas E., Escalante T., Arce V., Chaves E., Velásquez R., Lomonte B., Rojas G., Gutiérrez J.M. (2001). The venom of *Bothrops asper* from Guatemala: Toxic activities and neutralization by antivenoms. Toxicon.

[27] Villalta M., Sánchez A., Herrera M., Vargas M., Segura Á., Cerdas M., Estrada R., Gawarammana I., Keyler D.E., McWhorter K. (2016). Development of a new polyspecific antivenom for snakebite envenoming in Sri Lanka: Analysis of its preclinical efficacy as compared to a currently available antivenom. Toxicon.

[28] Gans I.M., Coffman J.A. (2021). Glucocorticoid-Mediated Developmental Programming of Vertebrate Stress Responsivity. Front. Physiol..

[29] Lambeau G., Gelb M.H. (2008). Biochemistry and physiology of mammalian secreted phospholipases A2. Annu. Rev. Biochem..

[30] Leiguez E., Zuliani J.P., Cianciarullo A.M., Fernandes C.M., Gutierrez J.M., Teixeira C. (2011). A group IIA-secreted phospholipase A2 from snake venom induces lipid body formation in macrophages: The roles of intracellular phospholipases A2 and distinct signaling pathways. J. Leukoc. Biol..

[31] Matysiak J., Dereziński P., Klupczyńska A., Matysiak J., Kaczmarek E., Kokot Z.J. (2014). Effects of a honeybee sting on the serum free amino acid profile in humans. PLoS ONE.

[32] Forni L.G., McKinnon W., Lord G.A., Treacher D.F., Peron J.-M.R., Hilton P.J. (2005). Circulating anions usually associated with the Krebs cycle in patients with metabolic acidosis. Crit. Care.

[33] Freitas M.A., Geno P.W., Sumner L.W., Cooke M.E., Hudiburg S.A., Ownby C.L., Kaiser I.I., Odell G.V. (1992). Citrate is a major component of snake venoms. Toxicon.

[34] Aird S.D. (2005). Taxonomic distribution and quantitative analysis of free purine and pyrimidine nucleosides in snake venoms. Comp. Biochem. Physiol. Part B Biochem. Mol. Biol..

[35] Caccin P., Pellegatti P., Fernandez J., Vono M., Cintra-Francischinelli M., Lomonte B., Gutiérrez J.M., Di Virgilio F., Montecucco C. (2013). Why myotoxin-containing snake venoms possess powerful nucleotidases?. Biochem. Biophys. Res. Commun..

[36] Kisiel W. (1979). Molecular properties of the Factor V-activating enzyme from Russell’s viper venom. J. Biol. Chem..

[37] Takeda S., Takeya H., Iwanaga S. (2012). Snake venom metalloproteinases: Structure, function and relevance to the mammalian ADAM/ADAMTS family proteins. Biochim. Biophys. Acta—Proteins Proteom..

[38] Takeya H., Nishida S., Miyata T., Kawada S., Saisaka Y., Morita T., Iwanaga S. (1992). Coagulation factor X activating enzyme from Russell’s viper venom (RVV-X). A novel metalloproteinase with disintegrin (platelet aggregation inhibitor)-like and C-type lectin-like domains. J. Biol. Chem..

[39] Loría G.D., Rucavado A., Kamiguti A.S., Theakston R.D.G., Fox J.W., Alape A., Gutiérrez J.M. (2003). Characterization of “basparin A,” a prothrombin-activating metalloproteinase, from the venom of the snake *Bothrops asper* that inhibits platelet aggregation and induces defibrination and thrombosis. Arch. Biochem. Biophys..

[40] Noutsos T., Currie B.J., Wijewickrama E.S., Isbister G.K. (2022). Snakebite Associated Thrombotic Microangiopathy and Recommendations for Clinical Practice. Toxins.

[41] Sunitha K., Hemshekhar M., Thushara R.M., Santhosh M.S., Sundaram M.S., Kemparaju K., Girish K.S. (2015). Inflammation and oxidative stress in viper bite: An insight within and beyond. Toxicon.

[42] Costa C.G., Guérand W.S., Struys E.A., Holwerda U., Brink H.J.T., De Almeida I.T., Duran M., Jakobs C. (2000). Quantitative analysis of urinary acylglycines for the diagnosis of beta-oxidation defects using GC-NCI-MS. J. Pharm. Biomed. Anal..

[43] Kand’ár R., Žáková P. (2008). Allantoin as a marker of oxidative stress in human erythrocytes. Clin. Chem. Lab. Med..

[44] Teixeira C., Cury Y., Moreira V., Picolo G., Chaves F. (2009). Inflammation induced by *Bothrops asper* venom. Toxicon.

[45] Tsugawa H., Ikeda K., Takahashi M., Satoh A., Mori Y., Uchino H., Okahashi N., Yamada Y., Tada I., Bonini P. (2020). A lipidome atlas in MS-DIAL 4. Nat. Biotechnol..

[46] Tsugawa H., Cajka T., Kind T., Ma Y., Higgins B., Ikeda K., Kanazawa M., Vandergheynst J., Fiehn O., Arita M. (2015). MS-DIAL: Data-independent MS/MS deconvolution for comprehensive metabolome analysis. Nat. Methods.

[47] Tsugawa H., Satoh A., Uchino H., Cajka T., Arita M., Arita M. (2019). Mass Spectrometry Data Repository Enhances Novel Metabolite Discoveries with Advances in Computational Metabolomics. Metabolites.

[48] Sumner L.W., Amberg A., Barrett D., Beale M.H., Beger R., Daykin C.A., Fan T.W.M., Fiehn O., Goodacre R., Griffin J.L. (2007). Proposed minimum reporting standards for chemical analysis Chemical Analysis Working Group (CAWG) Metabolomics Standards Initiative (MSI). Metabolomics.

[49] Dieterle F., Ross A., Schlotterbeck G., Senn H. (2006). Probabilistic quotient normalization as robust method to account for dilution of complex biological mixtures. Application in 1H NMR metabonomics. Anal. Chem..

[50] Rohart F., Gautier B., Singh A., Lê Cao K.A. (2017). mixOmics: An R package for ’omics feature selection and multiple data integration. PLoS Comput. Biol..

[51] Ritchie M.E., Phipson B., Wu D., Hu Y., Law C.W., Shi W., Smyth G.K. (2015). limma powers differential expression analyses for RNA-sequencing and microarray studies. Nucleic Acids Res..

[52] Pang Z., Chong J., Zhou G., De Lima Morais D.A., Chang L., Barrette M., Gauthier C., Jacques P.É., Li S., Xia J. (2021). MetaboAnalyst 5.0: Narrowing the gap between raw spectra and functional insights. Nucleic Acids Res..

[53] Pang Z., Zhou G., Ewald J., Chang L., Hacariz O., Basu N., Xia J. (2022). Using MetaboAnalyst 5.0 for LC-HRMS spectra processing, multi-omics integration and covariate adjustment of global metabolomics data. Nat. Protoc..

[54] Mohamed A., Molendijk J., Hill M.M. (2020). lipidr: A Software Tool for Data Mining and Analysis of Lipidomics Datasets. J. Proteome Res..

[55] Molenaar M.R., Jeucken A., Wassenaar T.A., Van De Lest C.H.A., Brouwers J.F., Helms J.B. (2019). LION/web: A web-based ontology enrichment tool for lipidomic data analysis. Gigascience.

