# Supplementary figures and images for: Longitudinal Metabolomics and Lipidomics Analyses Reveal Alterations Associated with Envenoming by Bothrops asper and Daboia russelii in an Experimental Murine Model
Source: Toxins (Basel). 2022 Sep 23;14(10):657. doi: 10.3390/toxins14100657 (PMC9610966; doi:10.3390/toxins14100657)

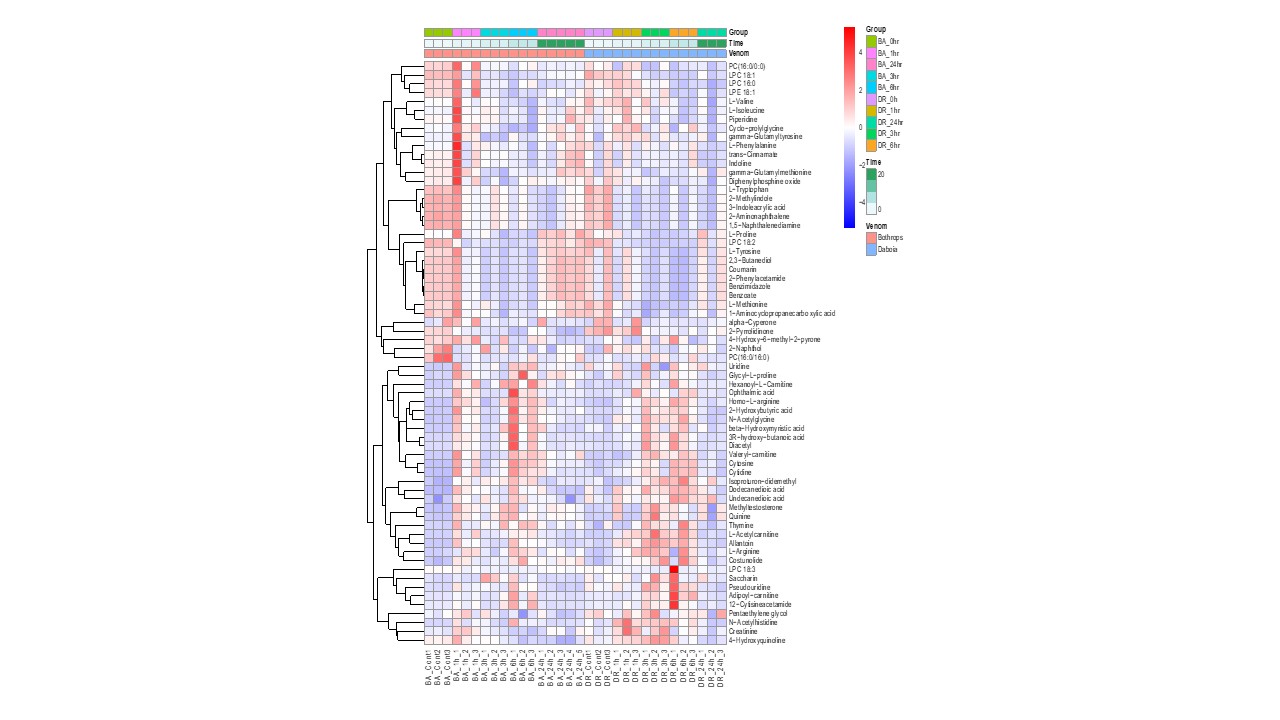

Supplement: Supplementary file 1 [file toxins-14-00657-s001.zip › Supplementary figure S1.jpg]

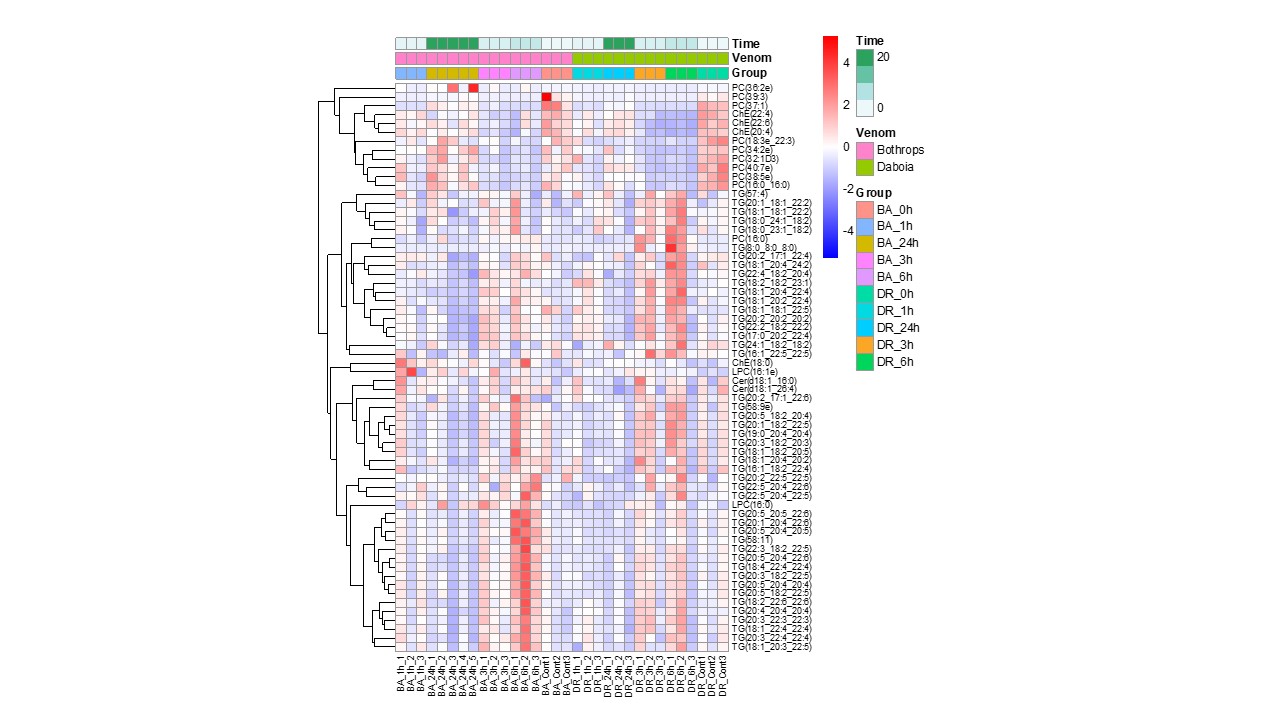

Supplement: Supplementary file 1 [file toxins-14-00657-s001.zip › Supplementary figure S2.jpg]
